# Leachate Pretreatment before Pipe Transportation: Reduction of Leachate Clogging Potential and Upgrading of Landfill Gas

**DOI:** 10.3390/ijerph19106349

**Published:** 2022-05-23

**Authors:** Mingde Xie, Xi Guo, Dan Liu

**Affiliations:** 1 Faculty of Geosciences and Environmental Engineering, Southwest Jiaotong University, Chengdu 610031, China; mingdexie@swjtu.edu.cn (M.X.); guoxisw@163.com (X.G.); 2 School of Life Science and Engineering, Southwest Jiaotong University, Chengdu 610031, China

**Keywords:** leachate, clogging, calcium, landfill gas, upgrading

## Abstract

Leachate and landfill gas are the main contaminants produced by modern sanitary landfills. The leachate easily leads to clogging in the leachate transportation pipe, and the landfill gas can be used as renewable energy after the removal of CO_2_. The study aims to investigate the removal of the major scale forming ion of Ca^2+^ in leachate using raw landfill gas before pipe transportation. The research demonstrated that, under the given experimental conditions, the removal rate of Ca^2+^ in the leachate was positively correlated with the pH value of the leachate, and negatively correlated with the intake flow rate of the landfill gas; the highest removal rate of Ca^2+^ was achieved when the intake flow rate and volume were 0.05 L/min and 2.0 L, respectively, and the highest removal rate of Ca^2+^ from the leachate was about 90%. The maximum removal rate of CO_2_ from landfill gas could reach 95%, and the CO_2_ content of the post-reaction gas was as low as 1.74% (volume percentage). The scanning electron microscope (SEM) and X-ray diffraction (XRD) analysis showed that the precipitate was spherical and mainly contained inorganic substances such as CaCO_3_, MgCO_3_, Ca(OH)_2_, Mg(OH)_2_, and SiO_2_. The study showed that, before the leachate was piped, the Ca^2+^ could be removed using the raw landfill gas, thereby reducing the potential for the formation of precipitation clogging in the pipeline. This study also provides new ideas for upgrading landfill gas to achieve a renewable-energy utilization plan, and reduces greenhouse gas emissions by reducing CO_2_ emissions from landfills.

## 1. Introduction

At present, sanitary landfill is one of the main methods of municipal solid waste (MSW) disposal in China [1,2,3]. The leachate produced by the landfill mainly consists of moisture contained in the landfill waste and the decomposition of organic matter in the waste [4]. Leachate is a high-concentration organic wastewater with a complex composition. If the leachate is not treated, it can contaminate groundwater and the surrounding environment [5]. In modern sanitary landfills, the leachate collection system (LCS) is the basic component located above the low-permeability liners at the bottom of the landfill. The leachate collection system transports the leachate into the regulating pool under gravity. Leachate needs to be piped to the leachate disposal plant for final treatment [6,7]. When the leachate transfer pipes have been in operation for a period of time, severe clogging often occurs in them [8,9,10,11].

The accumulation of clogging materials in the pipes is a biological, chemical, and physical interaction process [12,13,14]. The main clogging material is calcium carbonate precipitate (CaCO_3_(s)) [15]. The CaCO_3_(s) content of the clogging materials in leachate transportation pipes can reach up to 84% [16,17]. In the leachate transportation pipes, anaerobic microorganisms ferment volatile fatty acids (VFAs) for growth and reproduction, and the by-products are carbon dioxide (CO_2_) and methane (CH_4_). CO_2_ dissolved in leachate forms weak acid carbonic acid (H_2_CO_3_), which converts the acid–base system of leachate from medium strong acid (VFAs) to weak acid (carbonic acid) and raises the pH of leachate; simultaneously, the dissolved H_2_CO_3_ ionizes more into carbonate (CO_3_^2−^), which reacts with Ca^2+^ in the leachate to form calcium carbonate precipitate (CaCO_3_(s)) [18,19,20]. The CaCO_3_(s) precipitate is related to the concentration of Ca^2+^ and chemical oxygen demand (COD) in the leachate [15,21]. CaCO_3_(s) deposits in the inner wall of the transportation pipes, thus reducing the cross-sectional area of the pipes and causing clogging in severe cases [22,23].

How to control the clogging in transportation pipes of leachate is of increasing concern to landfill operators and researchers. One method is to flush out the clogging materials that are not firmly anchored to the inner wall of the pipes during the initial stages of clogging through high-pressure water jetting [16,17]. Regularly flushing the leachate transportation pipes is a passive treatment method. CaCO_3_(s) is the main leachate material that forms precipitation clogging in the pipeline [15,24]. Hence, an active pretreatment method is to remove Ca^2+^ from the leachate prior to pipeline transportation, thereby reducing the potential for the formation of precipitation clogging in the pipeline.

Landfill gas is a biogas mixture produced by municipal solid waste when anaerobic microorganisms decompose organic components. The main components are CH_4_ (40–60%) and CO_2_ (30–50%) [25,26,27,28]. The removal of CO_2_ greatly increases the quality and calorific value of landfill gas, and the remaining bio-methane can be used for renewable energy [29,30,31]. The methods for removing CO_2_ components in landfill gas mainly include chemical absorption [7,32], membrane separation [33], pressure swing adsorption [34], biological techniques [35], cryogenic separation, and water scrubbing [36]. The chemical absorption method is based on the fact that the CO_2_ component easily reacts with an alkaline solution (such as sodium hydroxide and potassium hydroxide) to form a carbonate solute. This removes the CO_2_ component from the landfill gas and then improves the heat value and utilization efficiency of landfill gas [29]. The use of a leachate adjusted to alkaline conditions as a chemical absorption solvent for landfill gas can not only remove CO_2_ from landfill gas and upgrade landfill gas [32], but Ca^2+^ from the leachate can also be removed. The CO_2_ greenhouse gas is fixed in the form of carbonate precipitate to achieve the environmental protection goal of carbon sinks and greenhouse gas emission reduction [37]. As CO_2_ dissolved in an alkaline solution reaches carbonate equilibrium (H_2_CO_3_* + HCO_3_^−^ + CO_3_^2−^), the three carbonates remain in equilibrium in the liquid phase, and the content of CO_3_^2−^ increases gradually when the pH is higher than 8.34 [38]; thus, raising the pH and intake landfill gas into the leachate promotes the reaction of Ca^2+^ with CO_3_^2−^ to form CaCO_3_(s), thereby removing Ca^2+^ in the leachate [10], and reducing the potential for the formation of precipitation clogging in the pipeline [20].

Previous studies using simulated landfill gas and (or) synthetic leachate to remove the Ca^2+^ in the leachate have shown that Ca^2+^ can be removed thoroughly by adjusting the simulated landfill gas flow rate, leachate pH, and temperature. One study found that, the smaller the landfill gas intake rate, the higher the pH of the leachate, the lower the system temperature, and the higher the removal rate of Ca^2+^ in the leachate [39]. The research using simulated landfill gas generally used a gas in which CO_2_ and N_2_ were mixed in a certain proportion, and the CO_2_ content was significantly different from the raw landfill gas [39]. Meanwhile, the purpose of using simulated landfill gas is relatively simple—it is only to investigate the effect of the CO_2_ component on the removal of Ca^2+^, and it is impossible to carry out the research on the purification and enrichment of the available energy CH_4_ component in landfill gas. The synthetic leachate used for clogging studies is mostly based on the physicochemical properties of young leachate, and is formulated using a variety of chemical reagents. The synthetic leachate contains higher VFAs and Ca^2+^, the pH is lower (at around 7.0), and the suspended solids (SS) content is low too [10,11,15]. During the formation of crystals of calcium carbonate, suspended solids in the raw leachate can act as a nucleus to promote the formation and precipitation of calcium carbonate precipitate [23]; however, as synthetic leachate does not contain suspended solids, research using it is insufficient. Therefore, studies using synthetic leachate and simulated landfill gas have some differences from raw leachate and landfill gas. In order to further study the feasibility of using raw landfill gas to prevent and control the clogging of leachate pipes and engineering applications to implement the technology, it is necessary to conduct research using raw leachate and landfill gas in landfills.

Herein, this study focused on using raw landfill gas to pretreat the leachate to remove the Ca^2+^ prior to pipe transportation by laboratory experiments. The optimal reaction conditions for the Ca^2+^ removal rate were obtained through a series of single-factor experiments, which were aimed at investigating the individual effects of the following operating factors: pH of the leachate, intake flow rate, and intake volume of raw landfill gas. The analysis of the chemical composition and morphology of the precipitate formed by X-ray diffraction (XRD), scanning electron microscopy (SEM), and evaluation of the landfill gas upgrade effect in this process was carried out. Basic research work was carried out to achieve the engineering purpose of using landfill gas to prevent and control the clogging of leachate pipe transportation and to use the new method of landfill gas upgrade.

## 2. Materials and Methods

### 2.1. Material and Column

In this study, the laboratory experiment was conducted in one of the municipal solid waste landfill sites in Chengdu, China. The leachate was taken from the landfill regulating pool, and the raw landfill gas was collected from the vertical gas guiding wells by nylon hose (with a gas flow meter, control valve, and aeration pump) from the landfill site.

The main properties of the raw leachate (A) and landfill gas (B) after long-term monitoring are shown in Table 1.

The leachate had a higher concentration of Ca^2+^ of 280–370 mg/L, and the pH was over 7; therefore, this was easy to clog through the precipitation of CaCO_3_(s) during transport in the pipe. The landfill gas flow rate of the vertical gas guide well used in this experiment was stable at 720 mL/min, while the volume percentage of CO_2_ was about 32–36%.

**Figure 1:**
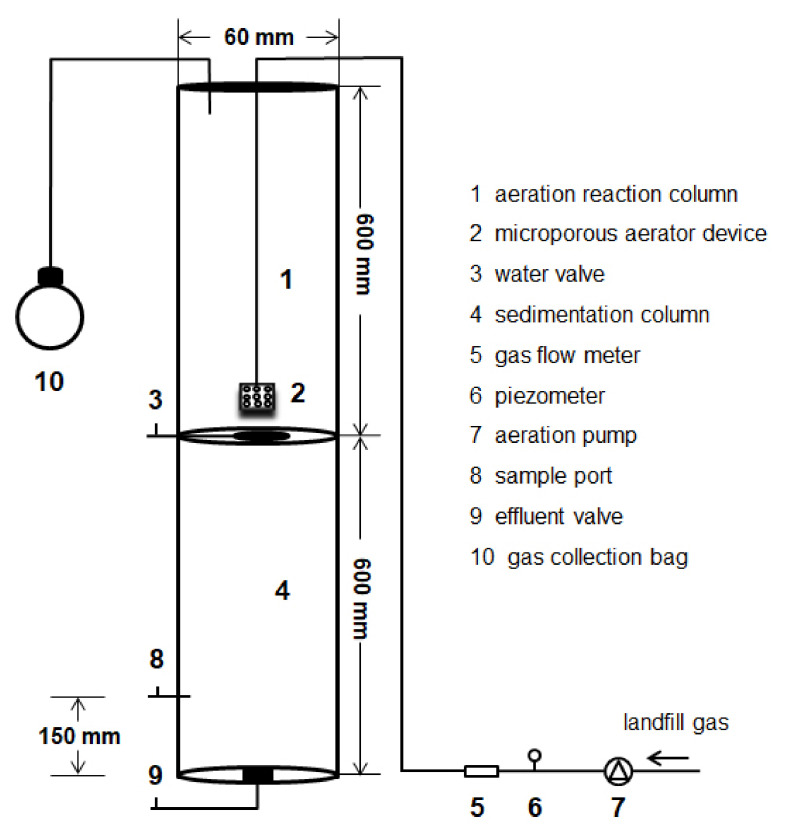
Column schematic (not to scale).

The experimental column (Figure 1) was made of a 10 mm thick acrylic tube. The device was mainly composed of an aeration reaction column (upper part of the experimental column), a sedimentation column (lower part of the experimental column), and a stainless-steel landfill gas intake pipe connected to a microporous aerator device located at the bottom of the aeration reaction column. The aeration reaction column and the precipitation column were 600 mm long. The effective length of the aeration reaction column was 500 mm, and the effective volume was 1.413 L. The leachate was added from the top of the aeration reaction column. The top of the experimental column was sealed with a rubber stopper, and two holes with a diameter of 5 mm were created (one for the intake port connecting the landfill gas intake pipe and one for the gas collection bag). A water valve was attached to the bottom plate of the aeration reaction column for discharging the leachate after the reaction into the sedimentation column for sedimentation and sampling. The landfill gas collected from the vertical gas pipe well was taken into the leachate by microporous aeration at the bottom of the aeration column. A leachate sample port was installed 150 mm above the bottom of the sidewall of the sedimentation column for the sampling analysis. The effluent valve was at the bottom of the sedimentation column.

### 2.2. Analytical Procedures

According to the experimental design, the pH of the leachate was adjusted by gradually adding sodium hydroxide (NaOH), and it was then injected into the aeration reaction column. A certain volume and flow rate of landfill gas was then introduced into the leachate through the microporous aerator device. The microporous aeration device can disperse the landfill gas introduced into fine bubbles, increase the surface area of the gas–liquid contact, and facilitate gas–liquid mass transfer. The leachate after the reaction was discharged into the sedimentation column through the water valve and settled for 60 min, and the supernatant was sampled and analyzed from the sampling port. The main analytical indicators included COD, Ca^2+^, Mg^2+^, and pH, among others. XRD and SEM analyses were performed on the precipitates obtained from the effluent port.

The COD concentration was measured using potassium dichromate titration. Ca^2+^ and Mg^2+^ were expelled by nitric acid and perchloric acid, and were then obtained using a z-5000 atomic absorption spectrometer (Hitachi, Japan). The total suspended solids (TSS) were tested using gravimetric measurement of the residue retained on a 0.45 µm glass fiber filter dried at 105 °C. The ammonia nitrogen concentration was analyzed by distillation neutralization titration. The pH was analyzed using a PHS-3C pH meter (Shanghai Precision Scientific Instrument Co., Ltd., Shanghai, China). The precipitate composition analysis was carried out on a dx-2700 XRD (Fangyuan Instrument Co., Ltd., Wenzhou, China), and the surface morphology was measured using a Quanta 250 SEM (FEI, Hillsboro, OR, USA). The landfill gas flow rate was measured with a gas flow meter, and the contents of CO_2_ and CH_4_ were measured using a GC7900 gas chromatograph (Shanghai Tianmei Instrument Co., Ltd., Shanghai, China). The detection methods of the main indicators are shown in Table 2.

The removal rate of Ca^2+^ (${\mathrm{Rt}}_{{\mathrm{Ca}}^{2+}}$) in the leachate was set as the difference between the content of Ca^2+^ in the influent (A_1_ mg/L) minus the content of the effluent (A_2_ mg/L) divided by the content of Ca^2+^ in the influent (A_1_) (Formula (1)).

(1) $${\mathrm{Rt}}_{{\mathrm{Ca}}^{2+}}=\left(A_{1}-A_{2}\right)/A_{1}$$

The CO_2_ removal rate (${\mathrm{Rt}}_{\mathrm{CO}2}$) was set as the difference between the content of CO_2_ in the intake gas (B_1_%) minus the content of the post-reaction gas (B_2_%) divided by the CO_2_ content of the intake gas (B_1_) (Formula (2)).

(2) $${\mathrm{Rt}}_{\mathrm{CO}2}=\left(B_{1}-B_{2}\right)/B_{1}$$

### 2.3. Main Experimental Methods

The effects of landfill gas intake volume, intake flow rate, and pH value of the leachate on the removal of Ca^2+^ were investigated. The design of each single-factor condition was as follows:

(1) Examining the volume of the landfill gas, the landfill gas intake flow rate was set at 0.05 L/min, and the leachate pH was adjusted to about 9.08, 9.63, 10.23, 10.71, and 11.50. The intake volumes were 0.5, 1.0, 1.5, 2.0, 2.5, and 3.0 L, respectively.
(2) Examining the intake flow rate of the landfill gas, the landfill gas volume was set at 2.0 L, and the pH of the leachate was adjusted to about 9.08, 9.63, 10.23, 10.71, and 11.50. The landfill gas intake flow rates were 0.05, 0.08, 0.1, 0.2, and 0.4 L/min, respectively.
(3) Examining the pH of the leachate, the landfill gas intake volume was set at 2.0 L, and the landfill gas intake flow rates were set at 0.05, 0.08, 0.1, 0.2, and 0.4 L/min. The leachate pH was adjusted to about 9.08, 9.63, 10.23, 10.71, and 11.50, respectively.

## 3. Results and Discussion

### 3.1. Effect of Volume of Landfill Gas Intake on the Rate of Calcium Removal

**Figure 2:**
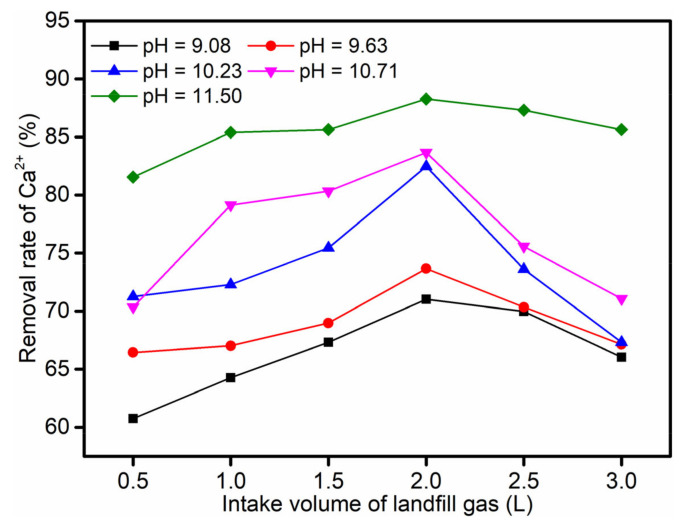
Effect of the intake volume on the rate of Ca^2+^ removal at different leachate pH levels.

When the landfill gas intake volume was less than 2.0 L, the removal rate of Ca^2+^ increased with the increase in the landfill gas volume; as it was more than 2.0 L, the removal rate of Ca^2+^ decreased. The removal rate of Ca^2+^ in the leachate reached a maximum at the landfill gas intake volume of 2.0 L at different leachate pH levels (Figure 2); when the leachate pH was set at about 9.08, 9.63, 10.23, 10.71, and 11.50, the maximum removal rates of Ca^2+^ were about 71.02%, 73.67%, 82.47%, 83.66%, and 88.27%, respectively.

When the landfill gas was taken into the leachate, the CO_2_ component dissolved and ionized to form bicarbonate (HCO_3_^−^) and carbonate (CO_3_^2^^−^) (Formulas (3)–(6)); with the gradual increase in the leachate pH value, the ionized carbonate also increased gradually, which reacted with Ca^2+^ in the leachate to form CaCO_3_(s) (Formula (7)). This may be the reason that the optimal intake volume corresponding to different pH values was 2.0 L. However, when the intake volume exceeded 2.0 L, the CO_2_ was passed into the leachate in excess, and the excess CO_2_ could react with the newly generated CaCO_3_(s) to form soluble Ca(HCO_3_)_2_ (Formula (8)). Hence, CaCO_3_(s) could be dissolved again [40]. Therefore, when the intake volume was much greater than 2.0 L, the excess CO_2_ could cause the Ca^2+^ removal rate in the leachate to decrease.

(3) $${\mathrm{CO}}_{2}\left(g\right)⇋{\mathrm{CO}}_{2}\left(\mathrm{aq}\right)$$

(4) $${\mathrm{CO}}_{2}+H_{2}O⇋H_{2}{\mathrm{CO}}_{3}$$

(5) $$H_{2}{\mathrm{CO}}_{3}⇋H^{+}+{\mathrm{HCO}}_{3}^{-}$$

(6) $${\mathrm{HCO}}_{3}^{-}⇋H^{+}+{\mathrm{CO}}_{3}^{2-}$$

(7) $${\mathrm{Ca}}^{2+}+{\mathrm{CO}}_{3}^{2-}⇋{\mathrm{CaCO}}_{3}↓$$

(8) $${\mathrm{CaCO}}_{3}+{\mathrm{CO}}_{2}+H_{2}O⇋\mathrm{Ca}{\left({\mathrm{HCO}}_{3}\right)}_{2}$$

### 3.2. Effect of Landfill Gas Intake Flow Rate and Leachate pH on the Removal Rate of Calcium

**Figure 3:**
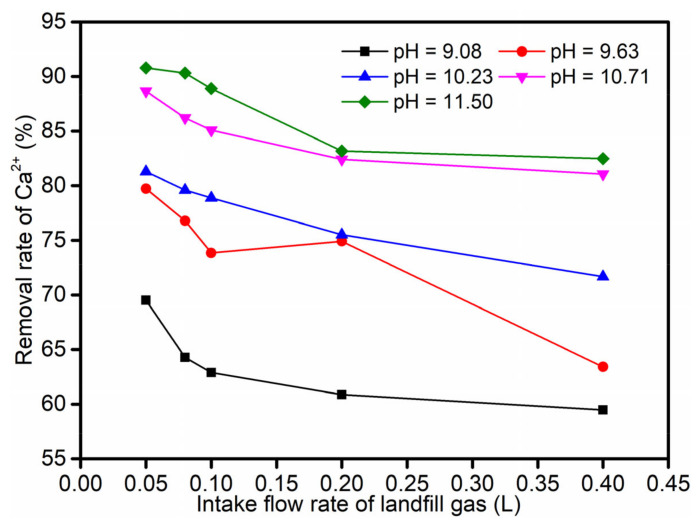
Effect of landfill gas intake flow rate and leachate pH on the removal rate of Ca^2+^.

Under all pH conditions, the removal rate of Ca^2+^ decreased with the increase in the intake flow rate of landfill gas (Figure 3). That is, the smaller the intake flow rate, the higher the removal rate of Ca^2+^ in the leachate. For the set experimental conditions, the optimal intake flow rate was 0.05 L/min, and the maximum removal rate of Ca^2+^ in the leachate was about 69.51–90.79%.

According to the double-membrane theory [41], after the adjustment of the pH of the leachate, the CO_2_(g) is easily dissolved and ionized into carbonate in the liquid phase. Therefore, the gas–liquid mass transfer of the landfill gas in the leachate is a type of gas-film control, and increasing the intake flow rate can increase the turbulence of the gas phase and increase the gas–liquid mass transfer rate. However, in the case when the intake volume is constant, the intake flow rate is increased and the intake time is shortened, so that the total absorption amount of the liquid relative to the CO_2_ component in the landfill gas is reduced. Therefore, an increase in the intake flow rate causes a decrease in the amount of CO_2_ absorption, so that the removal rate of Ca^2+^ in the leachate is lowered.

**Figure 4:**
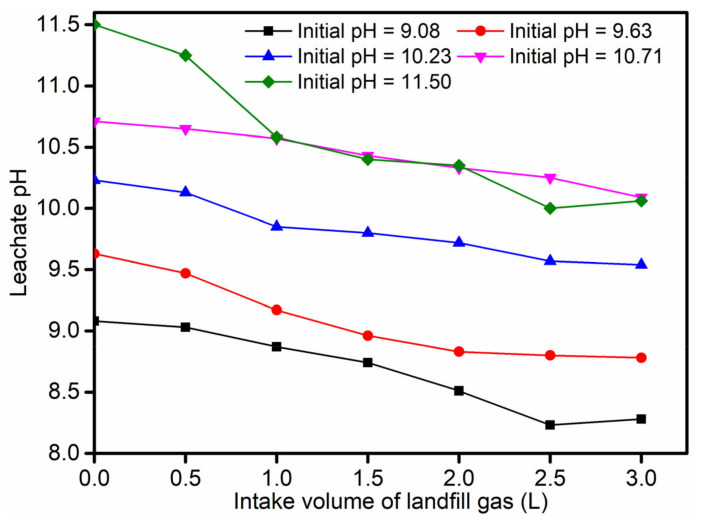
The intake flow rate of 0.05 L/min. The pH value of the leachate varies with different intake volumes of landfill gas.

As revealed in Figure 3, the removal rate of Ca^2+^ increased with the increase in pH from 9.08 to 11.5 at each intake flow rate. The landfill gas intake flow rate was 0.05 L/min; the leachate pH values were about 9.08, 9.63, 10.23, 10.71, and 11.50; and the corresponding Ca^2+^ removal rates were about 69.51%, 79.73%, 81.30%, 88.67%, and 90.79%, respectively. Thus, a satisfactory removal rate of about 90% could be achieved. At the same time, it could be observed that, when the pH was lower than 10.71, the removal rate of Ca^2+^ increased rapidly with the increase in pH value. When the pH was further increased, the effect on the removal rate of Ca^2+^ slowed down; at the intake flow rate of 0.05 L/min, the Ca^2+^ removal rate of leachate at pH 11.50 was not much different from that at pH 10.71. The partial pressure of CO_2_ in the landfill gas was relatively large; according to Henry’s law, more CO_2_ was dissolved in the leachate. When the CO_2_ in the landfill gas was taken into the leachate, part of CO_2_(g) dissolved in the leachate and converted to CO_2_(aq) (Formula (3)). CO_2_(aq) reacts with H_2_O to form carbonic acid (Formula (4)). Carbonic acid underwent primary and secondary ionization reactions to form carbonate equilibrium (Formulas (5) and (6)). According to the carbonic acid balance theory, the content of CO_3_^2^^−^ in the CO_2_-solution system increases with the increase inpH; especially in the range of 8.34 to 11, the CO_3_^2−^ content increases faster [39]. Therefore, the removal rate of Ca^2+^ increases as the pH of the leachate increases. When the pH is over 11, dissolved CO_2_ mainly exists in the form of CO_3_^2−^; at this time, the pH of the leachate increases again and the content of CO_3_^2−^ no longer significantly increases, and the pH has a smaller effect on the removal rate of Ca^2+^. Meanwhile, excessive Na^+^ is introduced into the leachate and the higher pH is not conducive to the subsequent leachate treatment [42,43]. The intake flow rate is 0.05 L/min, and the change trend of the leachate pH value under different intake volumes is shown in Figure 4. With the continuous introduction of landfill gas, the pH of the leachate gradually decreased, but remained alkaline. Therefore, in order to prevent the clogging of the leachate pipeline and facilitate the subsequent treatment of the leachate, the pH of the leachate can be controlled below 11. In this case, a better Ca^2+^ removal rate can still be achieved.

### 3.3. Characterizations of Precipitates

**Figure 5:**
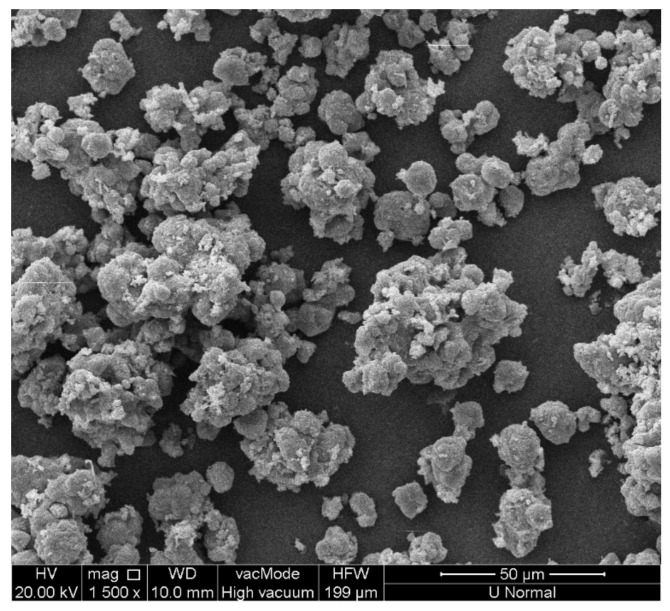
SEM of precipitates from the bottom of the sedimentation column.

**Figure 6:**
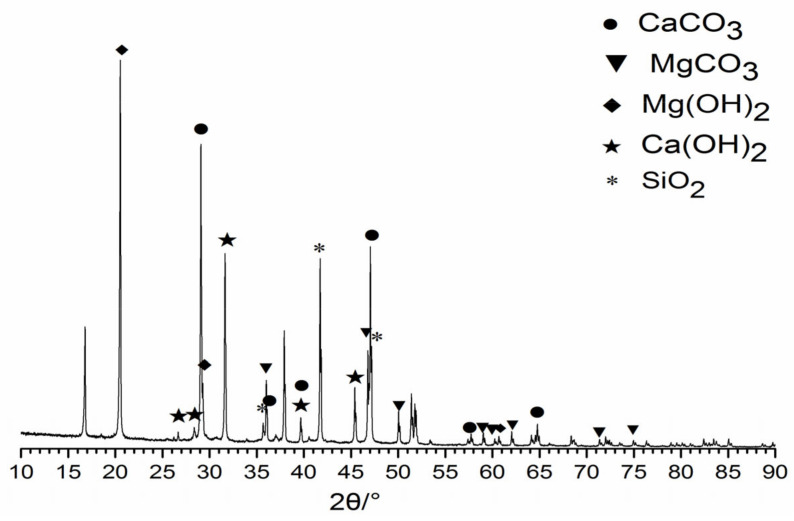
XRD of precipitates from the bottom of the sedimentation column.

The leachate discharged from the bottom of the sedimentation column contained a large amount of precipitates. The precipitates were granular (Figure 5). They could be deduced to be deposited easily at the bottom of the experimental column and engineering application facility, and so were easy to clean up. The main precipitates were CaCO_3_, MgCO_3_, Ca(OH)_2_, Mg(OH)_2_, and SiO_2_ (Figure 6).

The leachate contained a large amount of TSS, which could serve as a crystal nucleus to facilitate the carbonate that forms a crystalline precipitate [23,44]. Under the action of anaerobic microorganisms in the leachate, the leachate itself becomes alkaline and contains a relatively high concentration of bicarbonate (HCO_3_^−^) and carbonate (CO_3_^2^^−^) [10]. When the pH was adjusted with NaOH and raw landfill gas was introduced, a large amount of CO_3_^2^^−^ was produced (Formulas (3)–(6)), and Ca^2+^ and CO_3_^2−^ reacted to form CaCO_3_(s) in the leachate (Formula (7)). As Mg^2+^ and Ca^2+^ are the same type of A^2+^ ions, the solubility product of MgCO_3_ is greater than that of CaCO_3_, and the content of Mg^2+^ in the leachate was high, so when the Ca^2+^ content decreased to a certain value, Mg^2+^ could also form MgCO_3_(s) precipitate with CO_3_^2^^−^ (Formula (9)). At a higher pH, some Ca^2+^ and Mg^2+^ and excess OH^−^ formed Ca(OH)_2_(s) and Mg(OH)_2_(s) because of the high instantaneous content of OH^−^ (Formulas (10) and (11)). When the landfill gas was introduced, Ca(OH)_2_ and Mg(OH)_2_ reacted with CO_2_ to form more insoluble CaCO_3_(s) and MgCO_3_(s) precipitates (Formulas (12) and (13)), but the precipitate still contained some Ca(OH)_2_ and Mg(OH)_2_.

(9) $${\mathrm{Mg}}^{2+}+{\mathrm{CO}}_{3}^{2-}⇋{\mathrm{MgCO}}_{3}↓$$

(10) $${\mathrm{Ca}}^{2+}+{\mathrm{OH}}^{-}⇋\mathrm{Ca}{\left(\mathrm{OH}\right)}_{2}↓$$

(11) $${\mathrm{Mg}}^{2+}+{\mathrm{OH}}^{-}⇋\mathrm{Mg}{\left(\mathrm{OH}\right)}_{2}↓$$

(12) $$\mathrm{Ca}{\left(\mathrm{OH}\right)}_{2}+{\mathrm{CO}}_{2}⇋{\mathrm{CaCO}}_{3}↓+H_{2}O$$

(13) $$\mathrm{Mg}{\left(\mathrm{OH}\right)}_{2}+{\mathrm{CO}}_{2}⇋{\mathrm{MgCO}}_{3}↓+H_{2}O$$

Therefore, CaCO_3_(s), MgCO_3_(s), Ca(OH)_2_(s), and Mg(OH)_2_(s) were contained in the obtained precipitates. Because of the complex composition of the leachate, other types of solids were also contained in the precipitates, for example, silica (SiO_2_); SiO_2_ is mainly derived from suspended solids and silt particles in the leachate, and is one of the substances that form the physical clogging [14].

### 3.4. Analysis of CO_2_ and CH_4_ Content Changes in Landfill Gas

**Figure 7:**
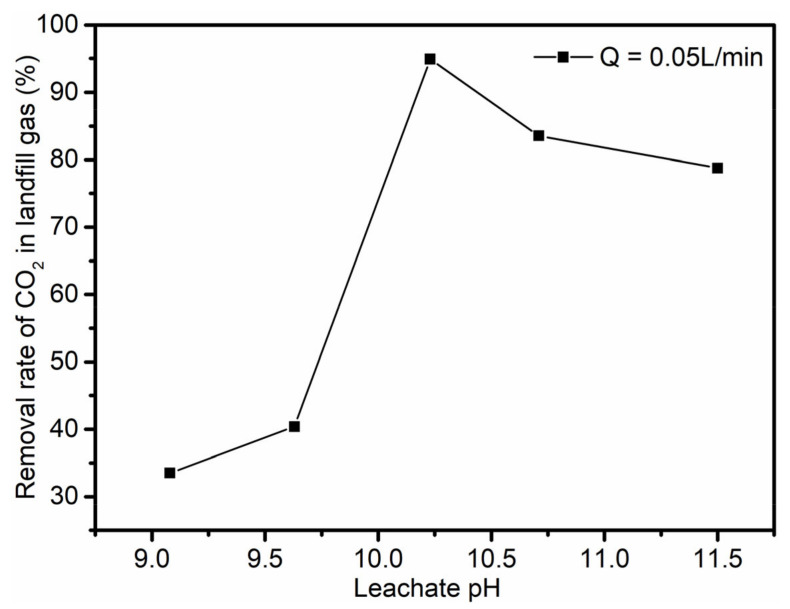
CO_2_ removal rate of landfill gas at different leachate pH levels.

The landfill gas intake flow rate was 0.05 L/min, the intake volume was 2.0 L, and the maximum removal rate of CO_2_ in the landfill gas was 95% (Figure 7). The CO_2_ content of the post-reaction gas was at least 1.74%, which meets the requirement that the CO_2_ component content of the landfill gas energy utilization rate not be higher than 3% [7].

**Figure 8:**
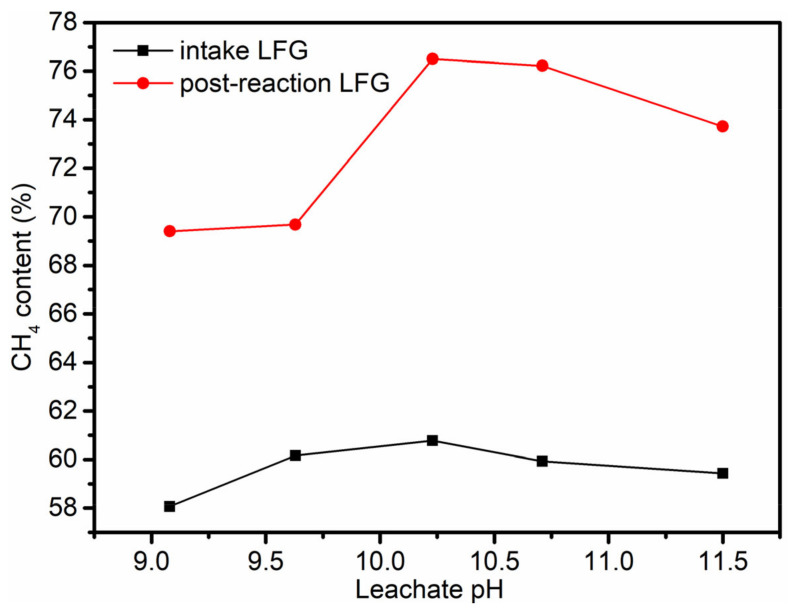
Changes in CH_4_ content in landfill gas before and after intake.

The removed CO_2_ component is fixed in the form of carbonate precipitations, which play a role in carbon emission reduction. The CH_4_ content of the post-reaction landfill gas was about 10–20% higher than that of the intake landfill gas, which is beneficial to the subsequent renewable energy utilization of landfill gas (Figure 8). As the pH of the leachate increased, it became more likely that the absorbed CO_2_ converted into CO_3_^2−^, which reacted with the Ca^2+^ in the leachate to form CaCO_3_(s), thereby promoting the absorption and reaction of CO_2_ in the leachate. The CO_2_ removal rate of the landfill gas increased with the pH of the leachate. Because the experiment was done in the field outside of the landfill site, the volume of leachate used in each experiment was so small that the leachate could not maintain a basically stable temperature, and the composition of the raw landfill gas also fluctuated within a certain range. This could affect the dissolution of CO_2_ in landfill gas, so some of the removal rates of CO_2_ fluctuated.

## 4. Conclusions

In this study, using raw landfill gas from the landfill site itself to remove Ca^2+^ in the leachate before pipe transportation was carried out to control the clogging. The optimum conditions were an intake flow rate of 0.05 L/min and intake volume of 2.0 L. To facilitate the subsequent treatment of the leachate, the leachate pH did not exceed 11. The optimal Ca^2+^ removal rate was about 90%, and the clogging could be controlled when the leachate was transported by pipe. The removal rate of CO_2_ in the landfill gas could increase up to 95%, and the CO_2_ content of the post-reaction gas reached a minimum of 1.74%. The CH_4_ content of the post-reaction landfill gas was about 10–20% higher than that of the intake landfill gas, which had important implications for the pretreatment and resource utilization of landfill gas.

The method of using CO_2_ from landfill gas to remove Ca^2+^ in the leachate presents the following environmental benefits.

(1) Most Ca^2+^ is removed prior to the leachate pipe transportation. This improves the service life of the relevant facilities and avoids the environmental pollution caused by the clogging of leachate pipes.
(2) The CO_2_ component of the landfill gas was mostly removed, and the upgrade of landfill gas increased its calorific value, laying the foundation for the energy utilization of landfill gas.
(3) CO_2_ is one of the main greenhouse gases, and landfills are one of its main sources; it is fixed by means of carbonate precipitation so as to reduce greenhouse gas emission.

Overall, it is completely feasible to use landfill gas to control the clogging of leachate, which is also in line with the policy of environmental protection and the comprehensive utilization of waste. 

This study has some shortcomings. First, the raw leachate used in the study was middle- or old-aged leachate, and in-depth research needs to be carried out for young leachate. Second, the use of sodium hydroxide to adjust the pH caused the leachate to contain more Na^+^, and the pH of the leachate after the reaction was still alkaline; these factors will adversely affect the subsequent treatment of the leachate. How to take into account both the control of the leachate to form clogging in the transportation pipeline and the convenience of the subsequent treatment of the leachate still needs to be further studied. Third, as our experiments were carried out on-site at the landfill site, we used raw leachate and landfill gas, which have certain fluctuations in their properties, so some measured data also fluctuated.

## Figures and Tables

**Table 1:** The main properties of the raw leachate and landfill gas.

(A) Raw Leachate	Concentration
pH	7.8–8.3
Chemical oxygen demand (mg/L)	2100–5700
Ammonia nitrogen (mg/L)	780–1130
Calcium (mg/L)	280–370
Magnesium (mg/L)	200–350
Total suspended solids (mg/L)	1000–3000
(B) Raw landfill gas	
CO_2_ (%)	32–36
CH_4_ (%)	52–62
Flow rate (mL/min)	720

**Table 2:** Analytical methods for the main indicators.

Indicators	Method
COD	potassium dichromate titration
Ca^2+^, Mg^2+^	atomic absorption spectrophotometry
TSS	gravimetric measurement
Ammonia nitrogen	distillation neutralization titration
pH	PHS-3C pH meter method
CO_2_, CH_4_	gas chromatography

## Data Availability

We chose to exclude this statement, because the study did not report any data.
